# Gamifying quantitative face-to-face interviews in rural India: An empirical evaluation based on the basic psychological needs theory

**DOI:** 10.1371/journal.pone.0244077

**Published:** 2021-01-28

**Authors:** Alice H. Aubert, Max N. D. Friedrich

**Affiliations:** 1 Eawag: Swiss Federal Institute of Aquatic Science and Technology, Dübendorf, Switzerland; 2 Ranas Ltd, Zurich, Switzerland; University of Milan, ITALY

## Abstract

**Objective:**

Standardized face-to-face interviews are widely used in low and middle-income countries to collect data for social science and health research. Such interviews can be long and tedious. In an attempt to improve the respondents’ experience of interviews, we developed a concept of gamified interview format by including a game element. Gamification is reported to increase engagement in tasks, but results from rigorously developed research are equivocal, and a theory of gamification is still needed.

**Materials & methods:**

We evaluated the proposed gamification with a randomized controlled trial based on self-determination theory, specifically on the basic psychological needs theory. In total, 1266 respondents were interviewed. Single and multiple mediation analyses were used to understand the effects of the gamified interview format.

**Results:**

Our evaluation showed that the gamification we had developed did not improve the outcome, the experience of the interview reported by respondent. The effect of the gamified interview format depended on the ability of respondents: gamification can be counterproductive if it overburdens the respondents. However, the basic psychological needs theory explained the mechanisms of action of gamification well: feeling competent and related to others improved the reported experience of the interview.

**Conclusion:**

We emphasize the need to develop context-specific gamification and invite researchers to conduct equivalently rigorous evaluations of gamification in future studies.

## 1. Introduction

### 1.1. General background and objectives

Standardized face-to-face interviews are one of the tools commonly used to collect data in both social science and health research [1]. In low- and middle-income countries, face-to-face interviews are the most common mode of data collection [2] because they sidestep sometimes low literacy rates, lack of reliable population data, and technological constraints. The sustainable development goals include Goal 3 “ensure healthy lives and promoting well-being for all at all ages” and Goal 6 “ensure availability and sustainable management of water and sanitation for all” [3]. Global efforts to achieve these increase the need for effective means of monitoring them, raising demand for face-to-face interviews. Depending on the complexity and length of the questionnaire, the skills of the data collectors, and the respondents’ abilities, respondents can easily become distracted, bored, and tired. This threatens the quality of data collected and may reduce the respondents’ willingness to participate in future surveys, which jeopardizes monitoring study results.

Concurrently, we observe a pervasive use of games or game elements in society [4], which has led to the neologism “gamification.” Various definitions of gamification exist, and researchers have attempted to theorize it (e.g. [5–9]). A common definition of gamification is “to use elements of game design in non-game contexts, products and services to motivate desired behaviors” [10]. Others see gamification as a process in which game elements, termed predictors, provide affordances that trigger psychological responses, labeled mediators, and consequently trigger behavioral responses, called outcomes [7, 11]. Gamification should lead to the internalization of extrinsic motivation [6], thus potentially enhancing respondents’ willingness to participate in surveys. Gamification has developed in several domains, such as human–computer interaction, and a recent review paper has suggested extending its study beyond this realm [9].

One of the domains increasingly using gamification is health and wellness studies. A search in the Web of Science Core Collection (retrieved on 26.07.2018) for articles and reviews published between 2002 and 2018 with the keywords “gamification” and “health” retrieved 93 publications, among which 51 featured “game” or “gamification” in their title. In this search, one publication dates from 2013, two from 2014, five from 2015, twenty-one from 2016, fourteen from 2017, and eight from 2018. Seven of them include the word “review” in their title. Health and exercise was also reported as the second largest category of empirical studies on gamification [9].

These review papers on gamification in the health domain suggest that further research is needed for the reasons highlighted here [12, 13]. Mixed results are reported about the effects of gamification. Gamification is sometimes successful when applied to increase motivation [14]. For instance, gamified cognitive tasks are more engaging and require less effort, resulting in lower drop-out rates from longitudinal studies than the traditional versions of the cognitive tasks [14]. However, including game mechanics can also lower performance in the task to be performed [14]. In addition, the evaluations of gamification suffer from small sample sizes [14], lack of control treatments [5, 9], and heterogeneous study designs [15, 16], for instance in the measures used. Some protocols for planned, structured, and standardized reviews have also been published [17], further indicating that the effectiveness and evaluation of gamification are open questions. Finally, some authors have called for randomized control trial testing of the use of single game elements [18].

Because interviews are time-limited processes, we thought that respondents might benefit from the short-term enhanced engagement and motivation triggered by gamification. This study develops and rigorously evaluates the effect and mechanisms of gamifying standardized face-to-face interviews with a randomized controlled trial. In particular, our study addresses **Research Question 1**: Do respondents evaluate the experience of the gamified interview format more positively than when it is not gamified?

The next sections present the design of the gamified interview format and the theoretical background used to evaluate it.

### 1.2 Theoretical background to evaluate the gamified interview format

A review of the literature indicates that self-determination theory is the psychological theory most frequently used in gamification studies [5, 19–23]. Most existing studies focus on the basic psychological needs theory (BPNT), one of the six mini-theories of self-determination theory, which is also used in the game and media research fields [24–26]. According to the BPNT, fulfilling or frustrating the three fundamental needs of competence (also termed mastery), autonomy, and relatedness influences well-being, deep and sustainable enjoyment of tasks, and thus motivation [27]. Our **Research Question 2** therefore asks: Is the effect of our gamification mediated through the variables of the basic psychological needs theory?

The BPNT considers three variables. Competence is described as “the need to feel effective and successful in the moment-to-moment activities of life” [6, 27]. Autonomy is “the need to feel volitional”: to have opportunities among which to choose. Relatedness is the need to feel part of society, supporting others who also support oneself. Any event can produce variations in the satisfaction or frustration of needs [27]. Autonomy supportive events facilitate satisfaction of the three needs, whereas controlling events tends to frustrate them [27]. Overall, autonomy, competence, and relatedness tend to relate positively to each other [27]. Designing gamification to fulfil these three fundamental needs and using game elements as events in nongame contexts enables internalization of the extrinsic motivation created by the game elements [6]. Improving the internalization of extrinsic motivation is a way to sustain the effect of gamification, as explained in Rigby’s motivational triad for gamification [6].

**Fig 1:**
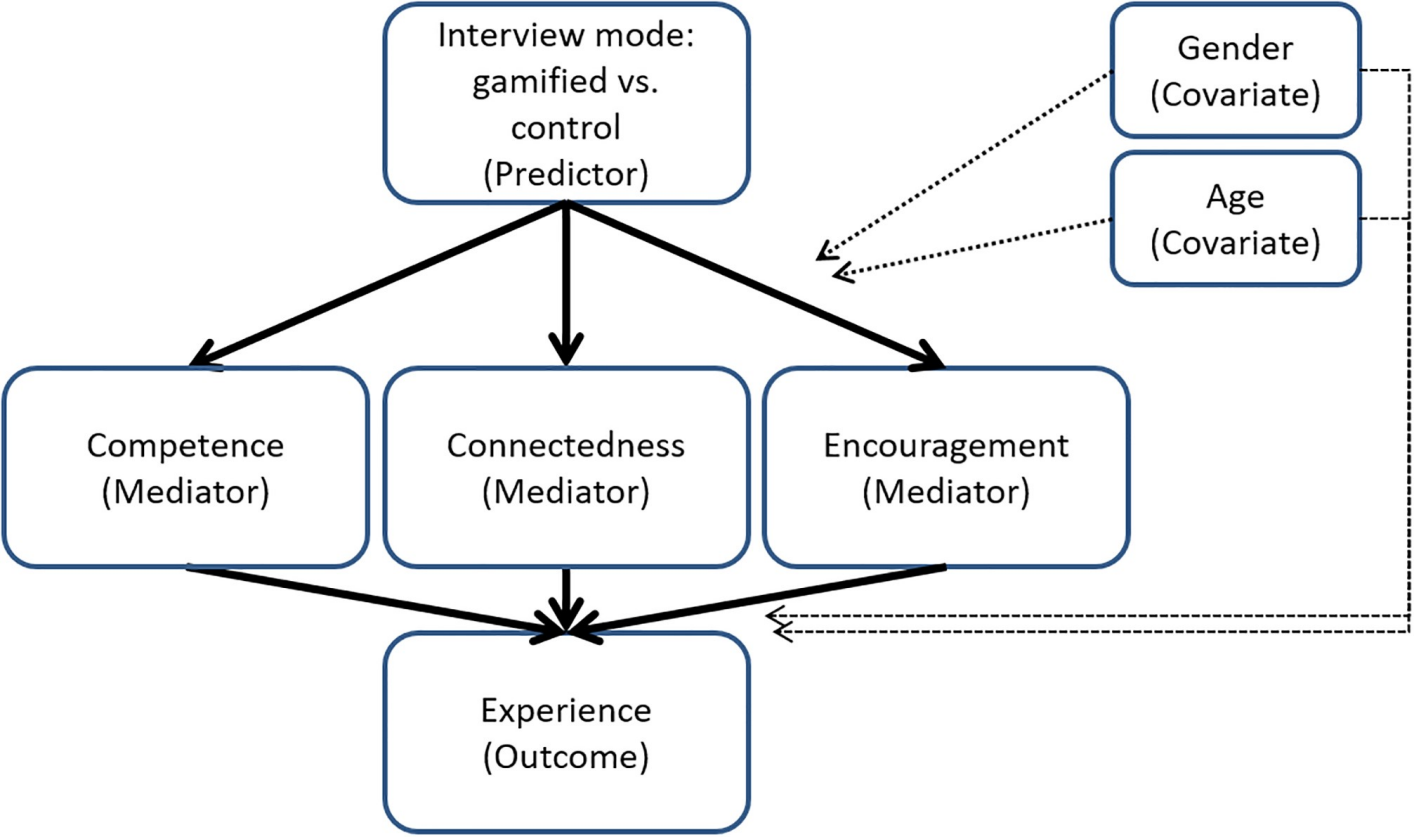
The initial conceptual model displaying the causal relationship based on the basic psychological needs theory. Relatedness is here divided into two dimensions, connectedness and encouragement. The intervention was not thought to increase autonomy, so this dimension is absent from this diagram.

We considered the following conceptual causal model for our gamified interview format (Fig 1). The predictor variable was the interview mode which was either gamified with a puzzle, as presented in Section 1.3, or the control condition. Our gamified interview format was designed to influence two psychological needs: competence and relatedness. In particular, we identified two dimensions for relatedness: connectedness between the respondent and the data collector and encouragement from the data collector. The satisfaction of these two basic psychological needs would mediate the effect of the interview mode on the perceived enjoyment, usefulness, and time consumption of the interview. In addition, we considered two design-relevant covariates, as suggested in the gamification literature [28]: gender and age.

### 1.3. Designing the gamified interview format

We developed the gamified interview format following Morschheuser et al.’s iterative, user-centered design process [29]: (1) project preparation to determine and prioritize the objectives of the gamification; (2) analysis of the context and of target group to define success criteria; (3) ideation to brainstorm ideas and consolidate some of them; (4) design of the prototype to be evaluated; (5) implementation of the gamification according to the results of the prototype test; (6) evaluation to test whether the implemented gamification fulfills the objectives it has been designed for; and (7) monitoring to continuously improve the solution. Our study consists of the evaluation of a prototype (step 6) and concludes with recommendations for Step 7. Steps 1 to 5 are briefly summarized here.

#### 1.3.1. Project preparation

We identified two objectives that the proposed gamified interview format should achieve, the more important of which was the first: (1) collect the answers to the psychological questionnaire about behavioral factors used to explain the variance in latrine use in households following the risks, attitudes, norms, abilities, and self-regulation (RANAS) model [30], and (2) make this individual interview of approximately 45 minutes’ duration more engaging for the respondent. The gamification focused most directly on the second objective.

#### 1.3.2. Analysis of context and users

The presented work was an add-on to the Promoting Latrine Use in Rural India Using the Risks, Attitudes, Norms, Abilities, and Self-regulation (RANAS) Approach to Systematic Behavior Change project [31]. In the main project, a 45-minute-long interview is conducted by a trained employee of a local data collection agency. This data collector asks the questions related to latrine use and the RANAS approach [30], and enters the answers directly into a database (using a specifically designed interface on a tablet). The targeted respondents are a rural population in South India. The overall population sample is large, with equal gender ratio, aged from 18 years old and no upper age limit. In the add-on study reported in this paper, we clearly focused on gamifying the interview and not on applying gamification to promote latrine use. Consequently, the gamification of the interview should be theme-independent and thus not connected to latrine use.

#### 1.3.3. Ideation

We brainstormed individually. First, we considered the three fundamental psychological needs specified in the BPNT (Section 1.2). We identified why they may not be fulfilled by the traditional interview procedure and how the interview could be modified to fulfil these needs. The gamification of the interview could increase feelings of (1) competence, adding the achievement of a parallel task and making the progress through the interview obvious, and (2) relatedness, triggering more interactions between respondent and data collector. We considered adapting existing games, such as the local game of carom, the happy families card game, and a puzzle game. The puzzle idea seemed the most practical and was selected. This idea was supported by some game studies that emphasize that jigsaw puzzles provide an achievable and fun experience that allows control over the process and is rewarding in itself [32]. This idea was submitted to the local Indian partners of the project, who were enthusiastic about it and validated our choice.

#### 1.3.4. Prototype

**Fig 2:**
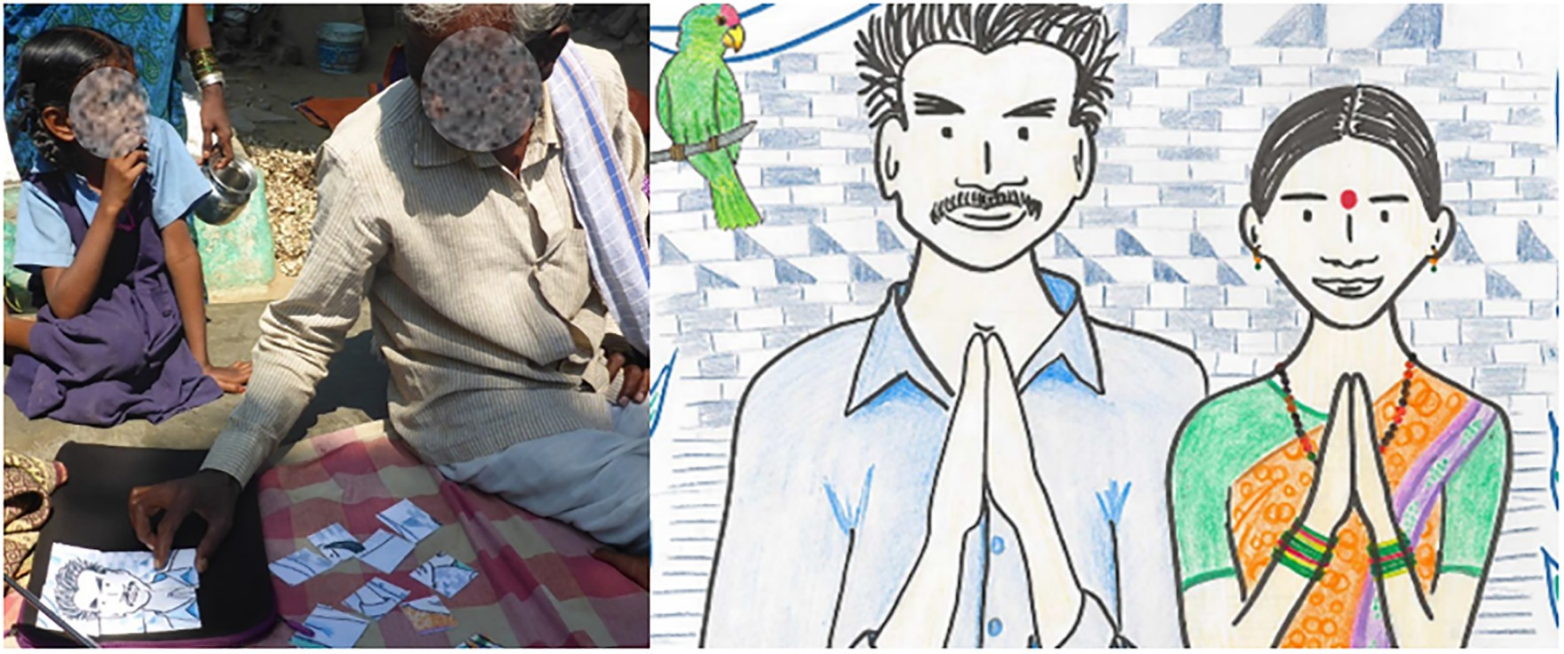
Prototype in the pretesting phase (left), and the final puzzle picture (right).

The puzzle should be completed along with the interview. This should increase the respondents’ feeling of competence while providing a sense of progress as the image appears [32]. The exchange of the puzzle pieces between the respondent and the data collector should increase their relatedness. The image of the puzzle should be independent of the topic, sanitation, because it should not be part of the intervention to promote latrine use. It needed to be neutral to target all participants without discrimination. We made a puzzle representing an Indian couple in local dress joining their hands in greeting (Fig 2, right). This matched the process: the complete image appeared at the end of the interview about latrine use and the RANAS approach, as the data collector thanked the participants. The image was depicted and emphasized the actual interview stage. It was color-printed on A4 cardboard and cut into 20 regular square pieces of similar size. After each interview section, the respondent received a piece of the puzzle.

#### 1.3.5. Prototype pretest

We pretested the puzzle in the field with the target respondents (Fig 2, left). We observed the respondents’ reactions when the data collectors introduced the puzzle and when they were doing the puzzle during the short interview breaks. Having completed the first pieces, most respondents seemed to enjoy adding other pieces to the puzzle. Although most respondents needed help placing the first pieces, most completed the puzzle independently after that. After the interview, we asked respondents about their experience with the puzzle. Most reported that they liked it. The data collectors were also debriefed after the interview. They reported that the puzzle was very positively received by the respondents. According to them, the puzzle served as an important tool to provide short breaks, opportunities for interaction, and a sense of progress during the interview.

#### 1.3.6. Implementation

The pretest showed that (1) most of the respondents seemed to enjoy the puzzle and (2) our target group did not know what a puzzle was before testing the prototype. Thus, we added a bird to the picture, which would appear on the first two pieces given to the respondent. It demonstrated to the respondent that each piece is part of a bigger picture. Some respondents doing a puzzle for a first time faced noticeable difficulties. This led to **Research Question 3**: Does the ability of respondents to complete the puzzle moderate the effect of the gamified interview format on the perceived experience and its mediators?

### 1.4. Formulation of hypotheses

The paper presents the evaluation of the gamification we proposed, structured according to the three research questions (RQs). We answered the research questions by testing the hypotheses presented in Table 1.

**Table 1:** Overview of the research questions (RQs) and hypotheses (Hs) considered in this study.

**RQ1**	**Do respondents evaluate the experience of the gamified interview format more positively than when it is not gamified?**		**Confirmed? (Summary of results)**
	H1a	The interview is evaluated as more enjoyable in the puzzle condition than in the control.	No
	H1b	The interview is evaluated as more useful in the puzzle condition than in the control.	No
	H1c	The interview is evaluated as less time-consuming in the puzzle condition than in the control.	No
**RQ2**	**Is the effect of our gamification mediated through the variables of the basic psychological needs theory?**		
	H2a	The puzzle has a significant positive effect on the feeling of competence compared to the control.	No
	H2b	The puzzle has a significant positive effect on the feeling of being connected with the data collector compared to the control.	No
	H2c	The puzzle has a significant positive effect on the feeling of being encouraged by the data collector compared to the control.	No
	H2d	Competence has a significant positive effect on evaluating the interview. H2da: more enjoyable, H2db: more useful, and H2dc: less time-consuming.	Partially
	H2e	Connectedness has a significant positive effect on evaluating the interview. H2ea: more enjoyable, H2eb: more useful, and H2ec: less time-consuming.	Partially
	H2f	Encouragement has a significant positive effect on evaluating the interview. H2fa: more enjoyable, H2fb: more useful, and H2fc: less time-consuming.	Partially
**RQ3**	**Does the ability of respondents to complete the puzzle moderate the effect of the gamified interview format on the perceived experience and its mediators?**		
	H3a to H3c	The ability of the respondent to complete the puzzle positively moderates the effect of the puzzle on competence, connectedness, and encouragement.	Partially
	H3d to H3f	The inability of the respondent to complete the puzzle negatively moderates the effect of the puzzle on competence, connectedness, and encouragement.	Partially

## 2. Data & methods

### 2.1. Trial design

This study was a randomized controlled trial (RCT) with one intervention arm and one nonintervention control. It was implemented in March and April 2018. There were no changes to the design after the trial commenced. The study is reported according to the CONSORT 2010 Statement: updated guidelines for reporting parallel group randomized trials [33]. This study was an add-on to the baseline survey of the Promoting Latrine Use in Rural India Using the Risks, Attitudes, Norms, Abilities and Self-regulation (RANAS) Approach to Systematic Behavior Change project [31]. The survey measured the psychosocial determinants of latrine use. The survey consisted of standardized face-to-face interviews on defecation practices and their behavioral determinants and constituted the baseline for a cluster-randomized controlled trial to evaluate interventions to promote latrine use. This study was approved by the institutional review board at the Faculty of Arts, University of Zurich on 18.09.2017, and all relevant permits were obtained from the Indian government. The study was registered at both the German Clinical Trials Register and the Registry for International Development Impact Evaluations under the IDs DRKS00013537 and 5a940c231baef, respectively.

### 2.2. Participants

This study was implemented in 120 villages in Raichur district, rural Karnataka, India, as described in more detail by Friedrich et al. [31]: Villages with at least 30% latrine coverage and at least 20 households owning a latrine were eligible for the study. The study villages were randomly selected from the 250 villages in Raichur district that fulfilled these criteria. Prior to the actual data collection, a household listing was conducted in all study villages. From this listing, 20 households were randomly selected from each village. Households with a functional latrine, defined as having at least a containment structure and squatting pan, were eligible for selection. Two respondents were selected from each household: one primary respondent and one back-up, to be surveyed if the first were unavailable. Household members of at least 18 years age were eligible. The interviews complied with the legal requirements in the region: each interview began only after the data collector had asked for the verbal informed consent of the respondent. Interviews were conducted through an international data collection agency. Data collectors had undergone a rigorous five-day theoretical and practical training program prior to the start of the study. The training was conducted by the data collection agency and the second authors of this study. It included a step-by-step explanation of the questionnaires and the underlying theory, a briefing about good field practices, group exercises, and role plays. Prior to the start of the actual survey, a two-day pretest was conducted. Data collectors asked the questions related to latrine use and the RANAS approach and entered the answers directly into a tablet computer using mWater software. Two data collectors were excluded from the survey team after the first interviews because their performance was deemed unsatisfactory by the field supervisor, in that they did not follow the correct consent procedure and did not ask the questions as specified in the questionnaire. Data collected by those data collectors was not included in the analysis.

### 2.3. Intervention

The intervention consisted of a jigsaw puzzle implemented alongside the standardized face-to-face interview on defecation practices and their behavioral determinants. See a detailed description in Section 1.3. After the first section of the interview, the data collector, initially asking the questions and entering the answers directly into a database, handed the first two puzzle pieces to the respondent and asked the respondent to fit the two pieces together. The data collector explained that the purpose of this game was to match each new piece to the existing pieces to create a picture. The data collector emphasized that the progress of the puzzle represented progress through the interview and that the interview was over when the puzzle was completed. After each of the 20 sections of the interview, the data collector handed over one piece of the puzzle to the respondent. The data collector paused the interview to allow the respondent to join the new piece to the existing pieces and look at the developing picture. After the final interview section, the final puzzle piece was handed over to the respondent, and the data collector asked the respondent how they liked the picture. In the control arm, the face-to-face interview on defecation practices and their behavioral determinants was conducted without the puzzle.

### 2.4. Measured variables

All outcomes were measured with a 5-minute face-to-face interview that was administered after the interview on latrine use was completed. The interview was conducted by the same trained data collectors who conducted the interview about defecation practices and applied the intervention for participants of that arm. Data were recorded on tablet computers using mWater software. The outcomes of this study captured how the respondents perceived the experience of the interview. The following items were used: “How enjoyable was it to answer this interview?”, “How useful for the community do you think this interview was?”, and “How time-consuming did you find this interview?”. The item measuring enjoyment was adapted from Ryan [24], and items measuring usefulness for the community and time consumption were adapted from Aubert and Lienert [34].

The measures for the mediators focused on competence and relatedness. The items were derived from the definition of competence and relatedness in Rigby [6]. Competence was measured with the following item: “How successful were you to answer the questions in a way that reflects your opinion?”. For Dimension 1 of relatedness, we used the item “How much connection did you feel between us?”, and for Dimension 2 “How much did you feel encouraged to answer the questions?”

Outcomes and mediators were measured using equidistant 5–point Likert scales. We used single-item measures given (1) the length of the whole interview; (2) the finding that additional items may bring little additional information [35].

We added a set of questions to be answered by the data collectors after the interview. These focused on the experience of the respondent as observed by the data collector. Following the pretest, we added a question about the ability of the respondent to complete the puzzle: “The puzzle seemed too hard for the respondent,” which could be answered with Yes, No, I do not know, or No puzzle.

### 2.5. Sample size

We defined a meaningful minimal effect to be half a scale-point difference in outcomes between intervention and control groups. We conducted a pilot data collection that yielded means of outcomes between 2.17 (SD = 1.27) and 3.71 (SD = 0.72). We assumed an alpha error probability of 0.05, which we corrected for testing six primary and secondary outcomes by dividing it by 6. Using g*power [36] and assuming a standard deviation of 1.3, statistical power of 0.8, and the corrected alpha error probability, we estimated that an effective sample size would require 145 respondents per group. Because the measurement of outcomes was to be conducted directly after the intervention, we did not expect any attrition or refusal. This study was nested in a larger longitudinal study (see explanation above), so the overall sample size was determined according to that study’s requirements. In addition, we wanted as many respondents as possible to benefit from the anticipated positive effect of gamification on interview experience. Therefore, we chose an unbalanced allocation of participants to the experimental conditions. This led to 1124 participants allocated to the intervention condition and 142 to the control condition.

### 2.6. Randomization

Respondents were assigned to intervention and control using blocked randomization, in which villages constituted the blocks. In each village, two respondents were randomly allocated to the control condition, and the remaining respondents were allocated to the puzzle condition. First, random numbers were generated using the RAND() function in Microsoft Excel, then households were sorted village-wise by the random number, and finally, the households with the highest random numbers were selected as intervention respondents. The second author enrolled the study respondents, generated the random numbers, and allocated respondents to control and intervention conditions.

### 2.7 Blinding

Because the intervention required active participation from both data collectors and respondents, blinding was not possible. Data collectors were blinded to the specific hypotheses. However, so that the puzzle could be implemented correctly, they were informed that the puzzle was added to the survey to allow a playful interaction between respondent and data collector and to visualize the progress through the interview.

### 2.8. Statistical methods

Statistical preliminary analyses (visualization of the data, Wilcoxon tests between groups) and data preparation were performed using R project for statistical computing [37]. We reversed the “time-consuming” item so that the assessments were all in the same direction. Then, we rescaled the 5–point Likert scale items from 0 to 1 to facilitate the interpretation of the regression coefficients.

To investigate hypotheses H1a to H1c, we conducted single mediation analyses, focusing on the direct effects, with the interview mode, puzzle or control, as predictor of each dimension of the experience. We performed the single moderation analyses using IBM SPSS Statistics 25 and the PROCESS macro (version 3.3) for SPSS [38, 39]. We estimated the 95% confidence intervals and used bootstrapping with 10,000 resamplings when those were estimated. The treatment was coded as a dummy variable with the interview without puzzle as control (0).

Hypotheses H2a to H2c were tested considering each a-path of multiple mediation analyses, also with the PROCESS macro in IBM SPSS Statistics 25 [38, 39]. The a-path corresponds to the influence of the predictor (i.e. interview mode, CON for the control condition, PUZ for the puzzle condition) on the mediator variable. Hypotheses H2d to H2f were tested considering each b-path of same multiple mediation analyses for each dimension of the experience. The b-path corresponds to the influence of the mediator variable (e.g. competence) on the outcome. Thus, we ran three multiple mediation analyses. The interview mode was the predictor; competence, connectedness, and encouragement were the mediators; and each dimension of experience was an outcome. Gender and age were included in the models as covariates.

To investigate hypotheses 3a–3f, we repeated the single and multiple mediation analyses as described above, but with different predictors. For H3a to H3c, we used the dummy variable created for the participants judged able to complete the puzzle (referred to as PUZ+, n = 947) as the predictor, and the dummy variable created for the participants judged unable to complete the puzzle (referred to as PUZ-, n = 177) as an additional covariate. For H3d to H3f, we used the dummy variable created for the participants judged unable to complete the puzzle (PUZ-) as the predictor and the dummy variable created for the participants judged able to complete the puzzle (PUZ+) as an additional covariate.

## 3. Results

In response to RQ 1, about the better evaluation of the experience of the interview with the proposed gamification than when it is not gamified, no statistically significant direct effect of the puzzle was detected on any outcome (a-path, e.g. in Table 2). Thus, H1a to H1c, about enjoyment, usefulness, and perceived low time consumption, respectively, were rejected.

**Table 2:** Simple causal mediation analysis for control vs. puzzle for each dimension of experience. The a-path corresponds to the influence of the predictor (i.e. interview mode) on the mediator variable. The b-path corresponds to the influence of the mediator variable (e.g. competence) on the outcome.

Experience		Enjoyable						Useful						Not time consuming					
	a-path	b-path	Indirect effects			Direct effects	Total effects	b-path	Indirect effects			Direct effects	Total effects	b-path	Indirect effects			Direct effects	Total effects
			LL	B	UL				LL	B	UL				LL	B	UL		
Competence	.02	.42***	-.01	.01	.02	-.01	-.00	.38***	-.01	.01	.02	-.01	.00	.02	-.00	.00	.00	.02	.02
Encouragement	-.00	.59***	-.02	-.00	.02	-.00	-.00	.44***	-.01	.00	.01	.00	.00	-.04°	-.00	.00	.00	.02	.02
Connectedness	.02	.28***	-.00	.01	.02	-.01	-.00	.16***	-.00	.00	.01	-.00	.00	-.04*	-.00	.00	.00	.02	.02

Note: Nonparametric bootstrap; CI with the percentile method 95%; simulations 10,000;

° *p* ≤ .10;

* *p* ≤ .05;

** *p* ≤ .01;

*** *p* ≤ .001. LL: lower limit, UL: upper limit.

In response to RQ 2, focusing on the mediation of the effect through the variables of the basic psychological needs theory, the effect of the puzzle on any of the mediator was not statistically significant and very small (a-path in Table 3). Thus, H2a to H2c were rejected on the positive effect of the puzzle on each of the three mediators considered: competence, being connected, being encouraged. However, competence, connectedness, and encouragement had relatively high positive and statistically significant effects on perceiving the interview enjoyable (b-path in Table 3: .10**, .11***, and.47***, respectively). These results supported H2da, H2ea, and H2fa. Encouragement and competence also had relatively high positive and statistically significant effects on perceiving the interview useful (.33***, and .20***, respectively). These results supported H2db to H2fb. Connectedness had a very small and statistically insignificant effect on perceiving the interview useful. H2eb was rejected. Competence had a very small positive but somewhat statistically significant (*p* ≤ .05) effect on perceiving the interview less time consuming (.08*). This supported H2dc. Connectedness and encouragement had very small negative but somewhat statistically significant (*p* ≤ .10) effects on perceiving the interview as less time consuming (-.04°, and -.06°, resp.), thus suggesting the interview was perceived as more time consuming. Thus, H2ec and H2fc were rejected.

**Table 3:** Multiple causal mediation analysis for control vs. puzzle for each dimension of experience. The a-path corresponds to the influence of the predictor (i.e. interview mode) on the mediator variable. The b-path corresponds to the influence of the mediator variable (e.g. competence) on the outcome.

Experience		Enjoyable					Useful				Not time consuming			
	a-path	b-path	Indirect effects				b-path	Indirect effects			b-path	Indirect effects		
		*R*^*2*^ = .*33*	LL	B		UL	*R*^*2*^ = .*24*	LL	B	UL	*R*^*2*^ = .*02*	LL	B	UL
Competence	.02	.10**	-.00	.00		.01	.20***	-.00	.00	.01	.08*	-.00	.00	.01
Encouragement	-.00	.47***	-.01	.00		.01	.33***	-.01	.00	.01	-.06°	-.00	.00	.00
Connectedness	.02	.11***	-.00	.00		.01	.01	-.00	.00	.00	-.04°	-.00	-.00	.00
Total indirect effects			-.01	.00		.02		-.01	.00	.01		-.00	.00	.00
Direct effects				-.01					-.00				.02	
Total effects				-.00					.00				.02	

Note: Nonparametric bootstrap; CI with the percentile method 95%; simulations 10,000;

° *p* ≤ .10;

* *p* ≤ .05;

** *p* ≤ .01;

*** *p* ≤ .001. LL: lower limit, UL: upper limit.

Results for RQ 3 are presented in Tables 4 to 7. The positive and statistically significant total effects in Tables 4 and 5 indicate that participants who were classified as being able to do the puzzle perceived the interview as more enjoyable, more useful, and less time-consuming than control participants did. According to the single mediation analyses, presented in Table 4, the effects of perceiving the interview as enjoyable and useful were mediated by competence and encouragement. However, these mediation effects were not corroborated by the multiple mediation analysis (Table 5). Hypotheses H3a and H3c are thus partly supported. As in the previous models, BPNT variables correlated with perceiving the interview as enjoyable and useful.

**Table 4:** Simple causal mediation analysis for control vs. able to do the puzzle (PUZ+) for each dimension of experience. The a-path corresponds to the influence of the predictor (i.e. interview mode) on the mediator variable. The b-path corresponds to the influence of the mediator variable (e.g. competence) on the outcome.

Experience		Enjoyable						Useful						Not time consuming					
	a-path	b-path	Indirect effects			Direct effects	Total effects	b-path	Indirect effects			Direct effects	Total effects	b-path	Indirect effects			Direct effects	Total effects
			LL	B	UL				LL	B	UL				LL	B	UL		
Competence	.07***	.42***	**.02**	**.03**	**.04**	.01	.04**	.38***	**.02**	**.03**	**.04**	.01	.04**	.00	.00	.00	.00	.04**	.04**
Encouragement	.04***	.59***	**.01**	**.02**	**.03**	.02*	.04**	.43***	**.01**	**.02**	**.03**	.03**	.04**	-.05*	-.01	.00	.00	.04**	.04**
Connectedness	.02	.28***	.00	.01	.01	.03**	.04**	.16***	.00	.00	.01	.04**	.04**	-.04*	.00	.00	.00	.04**	.04**

Note: Nonparametric bootstrap; CI with the percentile method 95%; simulations 10,000;

° *p* ≤ .10;

* *p* ≤ .05;

** *p* ≤ .01;

*** *p* ≤ .001. LL: lower limit, UL: upper limit.

**Table 5:** Multiple causal mediation analysis for control vs. able to do the puzzle (PUZ+) for each dimension of experience. The a-path corresponds to the influence of the predictor (i.e. interview mode) on the mediator variable. The b-path corresponds to the influence of the mediator variable (e.g. competence) on the outcome.

Experience		Enjoyable				Useful				Not time consuming			
	a-path	b-path	Indirect effects			b-path	Indirect effects			b-path	Indirect effects		
		*R*^*2*^ = .*34*	LL	B	UL	*R*^*2*^ = .*25*	LL	B	UL	*R*^*2*^ = .*03*	LL	B	UL
Competence	.03**	.09**	.00	.00	.01	.18***	.00	.01	.01	.05	-.00	.00	.01
Encouragement	.01	.47***	-.01	.00	.02	.33***	-.01	.00	.01	-.06*	-.00	-.00	.00
Connectedness	.03	.11***	-.00	.00	.01	.01	-.00	.00	.00	-.04°	-.00	-.00	.00
Total indirect effects			-.01	.01	.03		-.00	.01	.02		-.00	.00	.00
Direct effects				.00				.00				.02°	
Total effects				.01				.01				.02°	

Note: Nonparametric bootstrap; CI with the percentile method 95%; simulations 10,000;

° *p* ≤ .10;

* *p* ≤ .05;

** *p* ≤ .01;

*** *p* ≤ .001.

LL: lower limit, UL: upper limit. With age, gender, and PUZ- as covariate.

**Table 6:** Simple causal mediation analysis for control vs. not able to do the puzzle (PUZ-) for each dimension of experience. The a-path corresponds to the influence of the predictor (i.e. interview mode) on the mediator variable. The b-path corresponds to the influence of the mediator variable (e.g. competence) on the outcome.

Experience		Enjoyable						Useful						Not time consuming					
	a-path	b-path	Indirect effects			Direct effects	Total effects	b-path	Indirect effects			Direct effects	Total effects	b-path	Indirect effects			Direct effects	Total effects
			LL	B	UL				LL	B	UL				LL	B	UL		
Competence	-.10***	.41***	**-.06**	**-.04**	**-.03**	-.02°	-.07***	.37***	**-.05**	**-.04**	**-.03**	-.03*	-.06***	.00	-.01	.00	.01	-.06***	-.06***
Encouragement	-.06***	.59***	**-.05**	**-.03**	**-.02**	-.03**	-.07***	.43***	**-.04**	**-.02**	**-.01**	-.04***	-.06***	-.06*	.00	.00	.01	-.06***	-.06***
Connectedness	-.01	.28***	-.02	.00	.01	-.06***	-.07***	.16***	-.01	.00	.00	-.06***	-.06***	-.04*	.00	.00	.00	-.06***	-.06***

Note: Nonparametric bootstrap; CI with the percentile method 95%; simulations 10,000;

° *p* ≤ .10;

* *p* ≤ .05;

** *p* ≤ .01;

*** *p* ≤ .001. LL: lower limit, UL: upper limit.

**Table 7:** Multiple causal mediation analysis for control vs. not able to do the puzzle (PUZ-) for each dimension of experience. The a-path corresponds to the influence of the predictor (i.e. interview mode) on the mediator variable. The b-path corresponds to the influence of the mediator variable (e.g. competence) on the outcome.

Experience		Enjoyable					Useful				Not time consuming		
	a-path	b-path	Indirect effects			b-path	Indirect effects			b-path	Indirect effects		
		*R*^*2*^ = .*34*	LL	B	UL	*R*^*2*^ = .*25*	LL	B	UL	*R*^*2*^ = .*03*	LL	B	UL
Competence	-.07***	.09**	**-.01**	**-.01**	**-.00**	.18***	**-.02**	**-.01**	**-.01**	.05	-.01	-.00	.00
Encouragement	-.05**	.47***	**-.04**	**-.02**	**-.00**	.33***	**-.03**	**-.02**	**-.00**	-.06*	.00	.00	.01
Connectedness	-.01	.12***	-.01	.00	.01	.01	-.00	.00	.00	-.04°	-.00	.00	.00
Total indirect effects			**-.05**	**-.03**	**-.00**		**-.05**	**-.03**	**-.01**		-.01	-.00	.01
Direct effects				-.03°				-.03°				-.03°	
Total effects				-.06**				-.05**				-.03°	

Note: Nonparametric bootstrap; CI with the percentile method 95%; simulations 10,000;

° *p* ≤ .10;

* *p* ≤ .05;

** *p* ≤ .01;

*** *p* ≤ .001.

LL: lower limit, UL: upper limit. With age, gender, and PUZ+ as covariate.

The total effects in Tables 6 and 7 indicate that participants who were classified as not being able to do the puzzle perceived the interview as less enjoyable, less useful, and more time-consuming than control participants did. According to both single and multiple mediation analyses, the puzzle’s effect on perceiving the interview as less enjoyable and less useful was mediated by lower degrees of perceived competence and encouragement. However, connectedness neither correlated with participation in the puzzle nor mediated any of the effects on interview experience. Hypotheses H3d and H3f are thus partly supported.

As suggested by Landers et al. [7], we used age and gender as covariates for the multiple mediation model. Gender had a slight effect (-.02) on the mediator feeling encouraged independently of the interview mode (*p*-values = .044, .067, and .067 for CON vs. PUZ, CON vs. PUZ+, and CON vs. PUZ- respectively): women felt less encouraged than men. Gender also had an effect on the perception of the interview as time consuming (-.02, *p*-value = .056, .079, and .079, for CON vs. PUZ, CON vs. PUZ+, and CON vs. PUZ- respectively): women perceived the interview as more time consuming than men.

## 4. Discussion

Our study aimed at rigorously evaluating the gamification of a long face-to-face interview in rural India. We addressed three research questions. RQ 1: Do respondents evaluate the experience of the gamified interview format more positively than when it is not gamified? RQ 2: Is the effect of gamification mediated through the variables of the basic psychological needs theory? RQ 3: Does the ability of respondents to complete the puzzle moderate the effect of the gamified interview format on the perceived experience and its mediators? To answer these questions, we conducted a randomized controlled trial with a large sample size, thereby contributing to gamification research [7, 18]. A further salient aspect of the research design is its shift away from the common human–computer interaction lab experiment to analog gamification in the field in a nonwestern context.

### 4.1. Answers to the research questions

The results of the single and multiple mediation analyses only partially supported our initial hypotheses. Our results for RQ 1 showed that the respondents in gamified interviews did not experience a better interview than those in the non-gamified interviews.

Results addressing RQ 2 showed that the gamification that we proposed did not have much effect on feelings of competence, connectedness, or encouragement. Irrespective of the puzzle, competence, encouragement, and connectedness had a positive effect on enjoying the interview. Competence and encouragement also had a positive effect on perceiving the interview as useful. In contrast, connectedness and encouragement were related to perceiving the interview as more time-consuming. These results suggest that the BPNT explains the mechanisms of perceiving the experience of the interview well. This is in line with some previous studies that reported that neither competence nor intrinsic motivation were improved by the gamification of a task, although in their cases the outcomes were improved [19].

**Fig 3:**
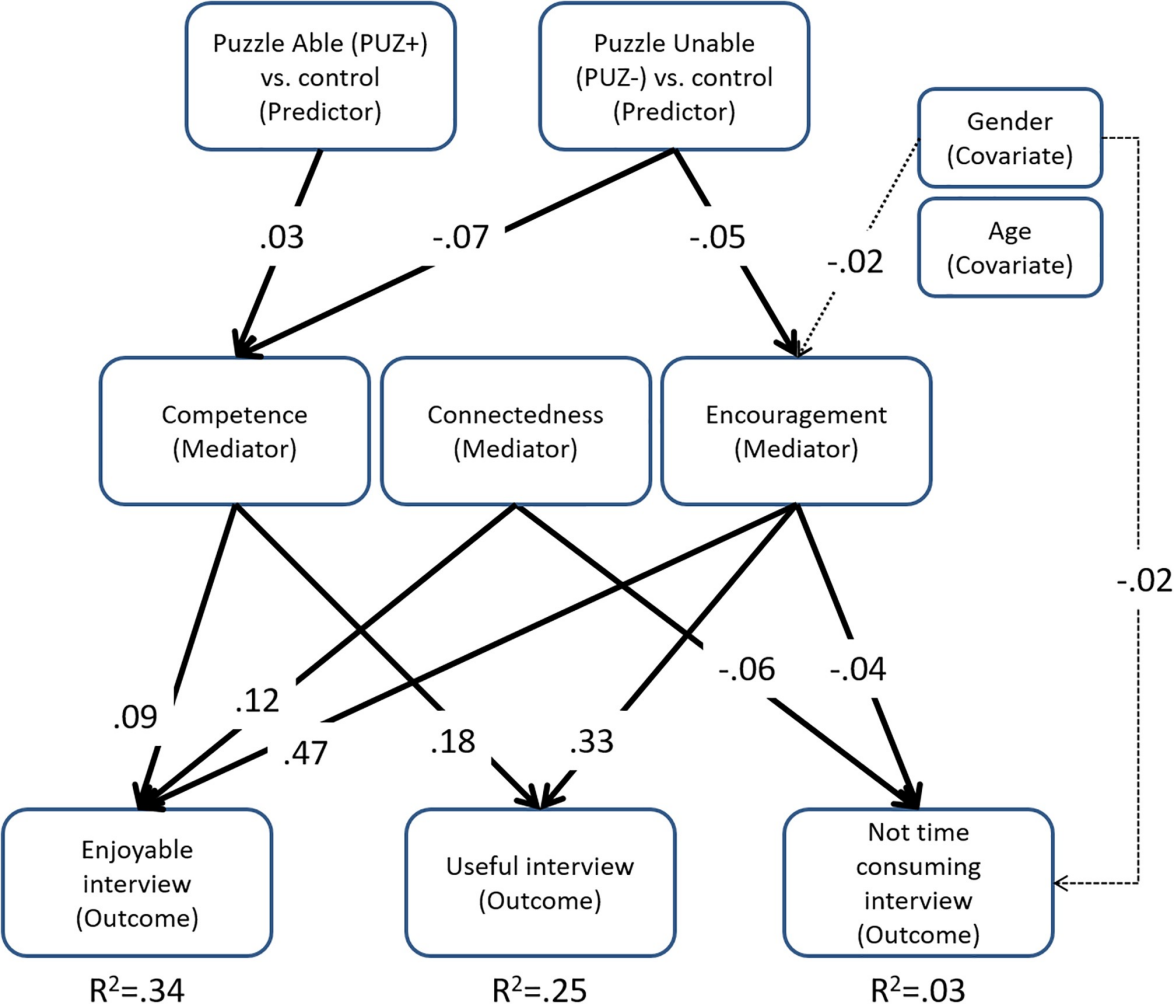
The final conceptual model displaying the statistically significant causal relationships (assessed by multiple mediation analysis, Tables 3, 5 and 7) based on the basic psychological needs theory.

In our results addressing RQ 3, we observed that the effects of our gamified interview format on the variables of the BPNT depended on the ability of the respondents to complete the puzzle. In particular, not being able to complete the puzzle had a negative effect on feelings of competence and encouragement, which in turn had a negative effect on the perception of the interview. Fig 3 summarizes the causal relationships that were statistically significant.

These results support three arguments. First, our rigorous evaluation indicated that the gamified interview format we tested did not improve outcomes. This was in line with a few studies on gamification and serious games in various fields reporting equivocal outcomes [15, 40]. Second, we found that the effect of the gamification we proposed depended on the ability of respondents to tackle it. When the respondents were not able to complete the puzzle, the outcome worsened. This echoed previous findings that including game mechanics can lower performance in the task to be achieved [14]. The cognitive load of the task should match the cognitive capacities of the targeted respondent. Finally, we confirmed earlier findings that the BPNT explains the mechanisms of action of gamification well [23–25].

### 4.2. Further research perspectives

The BPNT partly explained respondents’ perceptions of the interview. However, the total indirect effects were rather low, as were the a-paths. This suggests either that the gamification we proposed was poor or that the theory was not fully applicable to our application context. Addressing the limits of the study (Section 4.4, e.g. targeting autonomy, revising items) in further research would contribute to a theory of gamification, as called for by Landers et al. [7]. In particular, others stress the interplay between the three basic needs proposed in BPNT [21], for example by suggesting that feeling autonomous, which was not addressed in our gamification concept, is necessary to feeling competent [19].

Finally, if we were to repeat such an experiment, we would (1) measure the experience of the data collectors as well, (2) evaluate the quality of the responses, and (3) investigate whether the choice of the image depicted on the puzzle influenced the results. (1) Informal feedback from the data collectors on the effects of gamification was positive, yet the numerical analysis showed that gamification did not statistically significantly improve the respondents’ experience of the interviews. Measuring the data collectors’ experience of the interviews and testing whether gamification improved it would provide a valuable addition to the study. (2) Furthermore, in this study, we considered the experience of the interview as outcome. We considered gamification as an engaging task that through playfulness could improve self-disclosure, especially with embarrassing or emotionally charged themes. However, engagement in those gamified activities could potentially also influence attention and alter data collection. We invite future work to assess gamification a step further by also considering data reliability as an outcome. Gamifying tasks, such as interviews, is a challenge that may require trade-offs: Can gamification of interviews improve data reliability and mood and engagement at the same time? (3) Given the chosen gamification (i.e. using a puzzle), additional research questions emerge: How much does the chosen image impact the results about the experience of the interview? Would another image lead to different appreciation of the interview? These questions could be addressed in future work by adding several branches to the gamified treatment, and depicting a different image on the puzzle in each branch. Gamification of face-to-face interviews is emerging, and associated research opportunities are manifold and expanding.

### 4.3. Practical implications

In our case, 16% of the sampled respondents were judged unable to complete the puzzle. This means that a large majority of respondents were able to complete the puzzle. Despite this, we could not find consistent evidence that the puzzle improved any of the dimensions of the experience considerably. If one wishes to gamify such long, quantitative, face-to-face interviews, we recommend making the gamification achievable for all and easy in general by simplifying the design as much as possible. In our study, all the pieces were squares. A potential improvement could be to have fewer large uniform areas in the drawing and to give the pieces irregular shapes to provide extra cues to completing the puzzle. Furthermore, we would give the data collectors an additional instruction: if respondents really face difficulties with the puzzle, ask them whether they want to continue it. If not, the data collector could go on without the puzzle. If yes, the data collector could provide help with the puzzle. Furthermore, adding game elements that would provide a feeling of autonomy, the third variable of the BPNT, might be of interest. This would include creating an environment with choices offered to the respondents and more generally a context where they would feel willing to answer the interview. For instance, providing a challenge or a mission could create this feeling of volition.

The gamification design method proposed by Morschheuser et al. [29] was a valuable and constructive guideline, and we recommend it. In particular, it stresses the importance of adapting the gamification to the context. The iterative design, with pretest of the prototype, also proved extremely useful. We were able to adapt the material and procedure to the results of the prototype pretest. We would stress the importance of cultural considerations to designing effective gamification.

### 4.4. Limitations

This study has several limitations. First, neither data collectors nor participants could be blinded to the intervention condition, as the puzzle required active participation from both of them. It is thus possible that the awareness of the puzzle biased their responses. However, neither intervention nor control participants knew that they were participating in a controlled study. We thus do not expect control participants to have compared themselves with intervention participants. Consequently, we do not expect the unblinded design to be a major origin of bias. Second, the proportion of variance in experience, explained through the mediators, was small. This could be due to (1) not measuring the need for autonomy, because the gamified interview format that we designed was not thought to influence this construct; (2) proposing items to measure the constructs that might benefit from refinement; or (3) using single items to measure the constructs, because this can lead to somewhat lower reliability [35]. Third, we should emphasize that the design of the proposed gamification is only relevant in the case of structured interviews. In the cases of semi-structured interviews and conversations, another gamified interview format should be designed.

## 5. Conclusion

This paper focuses on an innovative use of gamification: for face-to-face interviews in a developing country. It reports on the development of a gamified interview format, its application in a real-world study, and its evaluation based on the basic psychological needs theory (BPNT), which is part of the self-determination theory. The rigorous evaluation showed that (1) the BPNT explained the mechanisms of action of gamification well, (2) the gamification that we designed did not improve the outcomes, and (3) the ability of the respondents to tackle the gamified interview format we designed was decisive to its effectiveness. This finding stresses the need for locally and culturally adapted gamification. We are also convinced that the research approach we used could be further developed in future research on gamification.

## Abbreviations

BPNT: basic psychological needs theory
CI: confidence interval
LL: lower limit
RANAS: risks, attitudes, norms, abilities, and self-regulation
RCT: randomized controlled trial
UL: upper limit
